# Transcriptome comparison reveals a genetic network regulating the lower temperature limit in fish

**DOI:** 10.1038/srep28952

**Published:** 2016-06-30

**Authors:** Peng Hu, Mingli Liu, Yimeng Liu, Jinfeng Wang, Dong Zhang, Hongbo Niu, Shouwen Jiang, Jian Wang, Dongsheng Zhang, Bingshe Han, Qianghua Xu, Liangbiao Chen

**Affiliations:** 1 Key Laboratory of Aquacultural Resources and Utilization, Ministry of Education, College of Fisheries and Life Sciences, Shanghai Ocean University, Shanghai, 201306, China; 2 Key Laboratory of Sustainable Exploitation of Oceanic Fisheries Resources, Ministry of Education, College of Marine Sciences, Shanghai Ocean University, Shanghai, 201306, China

## Abstract

Transcriptional plasticity is a major driver of phenotypic differences between species. The lower temperature limit (LTL), namely the lower end of survival temperature, is an important trait delimiting the geographical distribution of a species, however, the genetic mechanisms are poorly understood. We investigated the inter-species transcriptional diversification in cold responses between zebrafish *Danio rerio* and tilapia *Oreochromis niloticus*, which were reared at a common temperature (28 °C) but have distinct LTLs. We identified significant expressional divergence between the two species in the orthologous genes from gills when the temperature cooled to the LTL of tilapia (8 °C). Five KEGG pathways were found sequentially over-represented in the zebrafish/tilapia divergently expressed genes in the duration (12 hour) of 8 °C exposure, forming a signaling cascade from metabolic regulation to apoptosis via FoxO signaling. Consistently, we found differential progression of apoptosis in the gills of the two species in which zebrafish manifested a delayed and milder apoptotic phenotype than tilapia, corresponding with a lower LTL of zebrafish. We identified diverged expression in 25 apoptosis-related transcription factors between the two species which forms an interacting network with diverged factors involving the FoxO signaling and metabolic regulation. We propose a genetic network which regulates LTL in fishes.

Understanding the genetic basis for inter-species phenotypic variation is a long-standing goal in biology^1^,^2^. Variation in gene expression pattern plays a key role in the evolution of phenotypic variation^3^. Fishes vary in their phenotypes in terms of physiologies and lethal temperature ranges due to their wide distribution^4^. At temperatures approaching the lower temperature limit (LTL), the physiological functions of fish are severely disturbed, which ultimately results in mortality^5^. The differences in LTL among fish species provide rich genetic resources for exploring the contributions of gene expression patterns to the variation of LTL in fishes. Numerous studies have studied the gene expression patterns and the associated regulatory mechanisms in various fish models exposed to cold challenges^6^,^7^,^8^,^9^,^10^,^11^,^12^.

Fish experience cold stress when water temperature decreases to their LTL. Cold stress results in a cascade of physiological and behavioral responses, from hormone secretion (brain) to locomotor activity (muscle)^13^,^14^. Of the functional organs in fish, the gill occupies a central role in environmental adaptation due to its involvement in respiration, iono-/osmoregulation, acid-base balance and waste nitrogen excretion, contributing critically to physiological homeostasis under stress^15^; the gill is believed to play a prominent role in obligatory physiological responses because it directly interfaces with the water. Gills show great morphological plasticity in response to temperature changes in some fishes^16^,^17^. Therefore, gills could be a suitable system for studying gene expression variations that contribute to environmental stress response. Previous efforts have produced a rich understanding of transcriptional responses to low-temperature stimuli in the fish gill^18^,^19^. However, little is known about how gene was regulated in the gill related to the LTL in fishes of different thermal histories.

Comparative studies on a genome-wide scale have been widely used to analyze inter-species differences in the transcriptional responses^20^,^21^,^22^,^23^ and to dissect changes in regulatory sequences contributing to gene expression divergence between species^24^,^25^. With the availability of genome sequences for an increasing number of fish species, it is now possible to conduct comparative analysis to dissect the relative importance of changes in gene regulation to cold sensitivity in fishes. Some fish species have emerged as excellent model systems allowing for genetic dissection of phenotypic changes under stress conditions by virtue of their cosmopolitan availability, relative ease of culture and availability of advanced molecular manipulations (e.g., reverse genetics), such as zebrafish *Danio rerio*^26^, and tilapia *Oreochromis niloticus*^27^. Tilapia belongs to the Cichlid family and is an economically important farmed fish. Tilapia originates from the tropical and subtropical regions of Africa and is unable to survive at temperatures lower than 10 °C^28^,^29^. Mass mortality can occur during over-wintering, which causes heavy economic loss^30^. Compared with tilapia, zebrafish are broadly distributed over the Indian subcontinent, including northern India, Nepal and Bangladesh, and they experience a wider range of temperatures, from as low as 6 °C in winter to over 38 °C in summer^31^. The differences in LTL between these two fish species could be used to explore the adaptive biological processes that are important in low temperatures. In this study, we used laboratory-raised tilapia and zebrafish that were both reared at 28 °C for at least five generations. We undertook a comparison of tilapia and zebrafish in the transcriptional responses to the same cooling scheme. Here we define “differentially expressed genes” as those whose expression is regulated by cold temperature within a species, and “zebrafish/tilapia divergently expressed genes” as those whose expression pattern is different between the two fishes at a cold stimulation. We systematically identified zebrafish/tilapia divergently expressed genes when the fishes were exposed to 8 °C, the LTL of tilapia, from which over-represented pathways were identified. We found sequential occurrence of five over-represented KEGG pathways from metabolism to apoptosis in the duration of 12 h of cold exposure, signifying a signaling cascade leading to cell apoptosis in the gill when fishes facing the lethally cold stress. We experimentally demonstrated differential progression of apoptosis in the gills of zebrafish and tilapia, and found that the extent of apoptosis in the gills was positively correlated with the time course of organismal morbidity. We thus revealed a link between gene expression variation and LTL in fishes. We further identified the *cis*-regulatory elements and *trans*-factors attributable to the divergently expressed genes involved in apoptosis in these species, extending our understanding on the regulation of LTL in various fish species.

## Results

### Distinct cold responses in zebrafish and tilapia under the same cooling scheme

**Figure 1:**
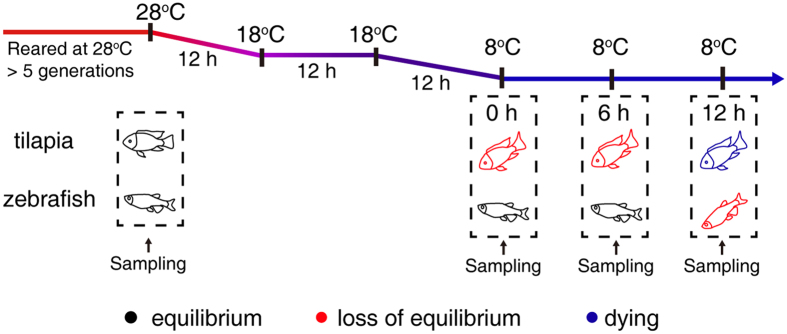
The changes in the body equilibrium of tilapia and zebrafish over time at 8 °C. Tilapia immediately lost body equilibrium when water temperature declined to 8 °C, whereas zebrafish maintained body equilibrium for the first 12 h at 8 °C. Twelve hours after loss of equilibrium, tilapia died, whereas zebrafish began to show equilibrium loss. The different body postures are indicated by different colors: black for normal equilibrium, red for loss of equilibrium, and blue for death. The sampling points are indicated by the arrows.

Both zebrafish and tilapia are reared in the laboratory at a stable temperature of 28 °C for at least five generations and are maintained in this temperature for the entire life prior to experiment. Individual zebrafish and tilapia were exposed to the same continuous, linear cooling scheme with water temperature declining from 28 °C to 18 °C and then to 8 °C (Fig. 1). The two species exhibited distinct LTLs. Zebrafish maintained body equilibrium for at least 10 h at 8 °C, whereas tilapia fell into a coma shortly after being exposed to 8 °C and died around 12 h. The course of body posture change in the two species was shown in Fig. 1. Although variations exist in the timing of equilibrium loss and death among individuals of a species, intra-specific variation ranged within half an hour for each of the time points denoted in Fig. 1. To minimize sampling variations, we decided to sample the tissues based on a scheme of predetermined time points. We chose three time points, 0 h, 6 h and 12 h, at 8 °C, representing the different phenotypic responses of the two species, for conducting inter-species comparisons using RNA-Seq. The sequencing information of a total of 24 samples (2 species × 3 time points × 3 biological replicates + 3 control samples from 28 °C) are listed in Table S1. Gene profiles of the replicates from the same temperature/time point were highly consistent (average Pearson correlation *r *= 0.96 and 0.95 for zebrafish and tilapia, respectively [Fig. S1]).

### Cold stress induces different expression patterns in gills between zebrafish and tilapia

**Figure 2:**
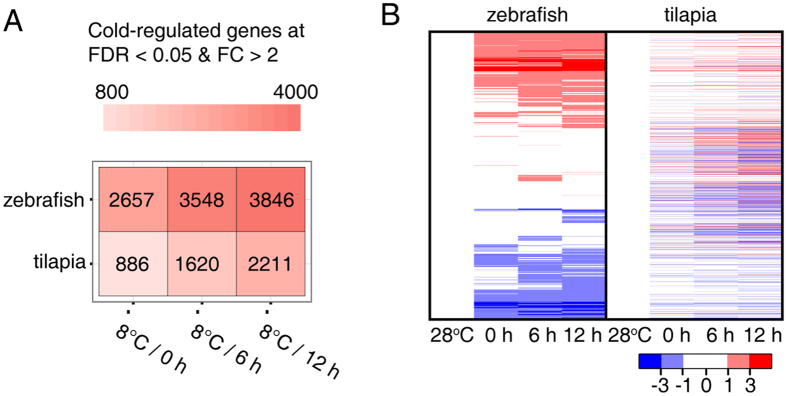
Global overview of differentially expressed genes at cold temperature in zebrafish and tilapia gills. (**A**) The differentially expressed genes at three time points of 8 °C exposure identified by comparing with the 28 °C control fishes. (**B**) Clustered profiling of 3,174 differentially one-to-one orthologous genes in zebrafish (left panel) and tilapia (right panel) based on comparisons between orthologous gene pairs. Log_2_ transformation of gene fold induction is indicated by the color-coded scales.

The differentially expressed genes at the cold temperature were identified by setting a criterion of >2-fold change in expression level (FDR < 0.05) compared with the control temperature (28 °C) in the two fish species. The number of differentially expressed genes was shown in Fig. 2A. The differentially expressed genes comprised 17.5% (4,838/27,635) and 13.4% (2,722/20344) of the expressed genes in zebrafish and tilapia, respectively (Table S2). The number of cold-induced genes in both species increased with prolonged cold treatment, but the extent of increase was different in the two species. The number of cold-induced genes identified at the 12 h time point was 2.5-fold (2,211/886) greater than that identified at 0 h in tilapia, whereas it only increased by 1.4-fold (3,848/2,657) in zebrafish when compared between the same time points (Fig. 2A).

To capture the differences in individual genes between the species, we first identified 12,072 expressed one-to-one orthologues between tilapia and zebrafish based on the defined orthologous groups from BioMart in Ensembl. We identified 3,174 one-to-one orthologous genes that were differentially expressed at the cold temperature either in zebrafish or tilapia. We compared the expression patterns of these 3,174 orthologues and found that they exhibited different profiles between zebrafish and tilapia (Fig. 2B). These findings indicate that extensive expression variation exists between zebrafish and tilapia related to their distinct LTLs.

### Functional divergence in zebrafish and tilapia gills identified from diverged gene expression patterns

**Figure 3:**
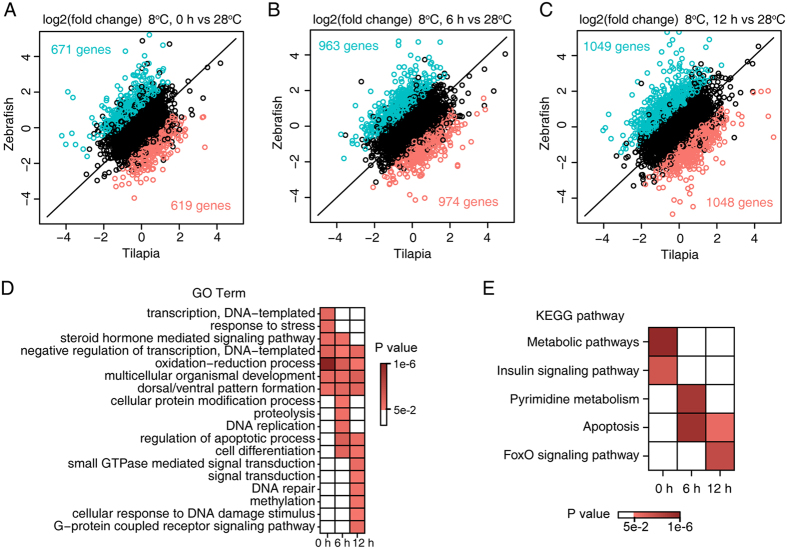
Divergent gene expression between zebrafish and tilapia in the LTL of tilapia. 1,290, 1,937 and 2,097 genes zebrafish/tilapia divergently expressed genes were identified at 0 h (**A**), 6 h (**B**) and 12 h (**C**), respectively. Green and red dots represent genes that are expressed in higher levels in zebrafish and tilapia respectively. (**D,E**) Heat map showing the enriched GO categories (**D**) and the KEGG pathways (**E**) identified from the zebrafish/tilapia divergently expressed genes at the three time points. The color scales depict the P values for the enrichment test, and cells in grey indicate a P value of >0.05.

To further investigate the expression divergence of one-to-one orthologous genes between species, we used a generalized linear model (GLM) approach (with variables of species and time, see Methods) to identify divergently expressed orthologous genes (defined as “zebrafish/tilapia divergently expressed genes”) in the time points examined. We identified 1,290 (10.7%), 1,937 (16.0%) and 2,097 (17.4%) zebrafish/tilapia divergently expressed genes at 0 h, 6 h and 12 h, respectively, between species (Fig. 3A–C and Table S3). A total of 2,848 zebrafish/tilapia divergently expressed genes were identified (Table S3). The zebrafish/tilapia divergently expressed orthologous genes were then used for gene ontology (GO) enrichment analysis to identify the over-represented biological functions or processes in the three time points. We identified 18 enriched GO terms (Fig. 3D). The enriched GO categories clearly characterized the species-specific responses of the two fishes to cold stress at different time points. Seven GO terms, including “DNA templated transcription”, “response to stress”, were enriched at 0 h. “Cellular protein modification process”, “DNA replication” and others were found to be enriched at 6 h, and GO terms including “small GTPase mediated signaling transduction”, “methylation”, “cellular response to DNA damage stimulus” and “DNA repair” were initially enriched at 12 h. Besides the aforementioned GO terms, several GO categories, such as “negative regulation of transcription” and “oxidation-reduction process” are enriched at all three time points and “regulation of apoptotic process” at 6 h and 12 h.

The divergently expressed genes in each time point were then used for identifying enriched KEGG pathways. Five pathways were found to be differentially regulated between the two species in the lethally cold exposure (Fig. 3E). Metabolic pathways, Insulin signaling pathway were enriched at 0 h, Pyrimidine metabolism and apoptosis were enriched at 6 h, while apoptosis and FoxO signaling pathway at 12 h. The time-lapse series of enriched GO terms and KEGG pathways clearly indicated progression of the differences in cold responsive programs between the two species.

### The Interplay between the enriched KEGG pathways

**Figure 4:**
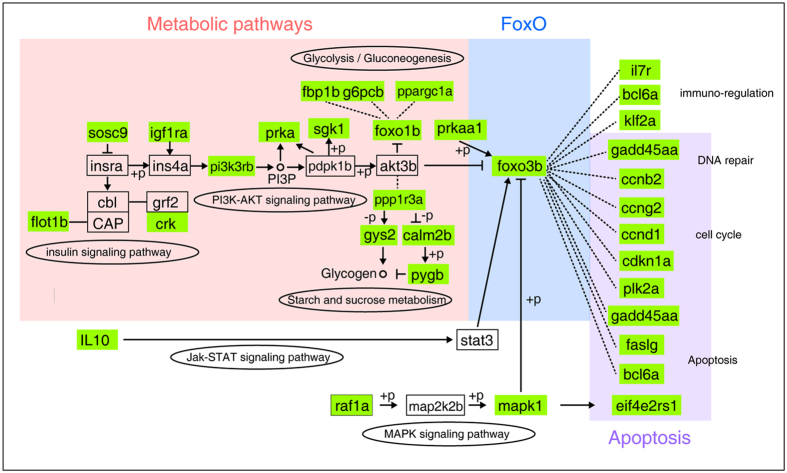
The relationship of the over-represented KEGG pathways inferred from regulatory networking of the essential factors of these pathways based on the established FoxO signaling pathway^57^. The zebrafish/tilapia divergently expressed genes were highlighted by blue color. The three pathways identified as over-represented in Fig. 3B were shadowed.

Given the phased occurrence of the over-represented KEGG pathways in facing the LTL exposure, it is likely that these pathways are interconnected in regulation. It is noticed that the FoxO signaling pathway is regulated by metabolic pathways^32^ and FoxO transcription factors play important roles in maintaining tissue homeostasis by regulating cell survival and apoptosis^33^. We thus constructed an interacting paradigm based on the well-established FoxO signaling pathway^34^, which was shown in Fig. 4. In this interacting network, thirty-three zebrafish/tilapia divergently expressed genes are involved with the enriched KEGG pathways, such as those belonging to the metabolism and apoptosis regulation pathways. These pathways are linked by components from the FoxO signaling pathway, such as Foxo3b, which transduces regulatory signals from the metabolic pathways to the downstream biological processes, including DNA repair, cell cycle and apoptosis. The interacting network of the zebrafish/tilapia divergently expressed genes implied a likely differential regulation of apoptosis between zebrafish and tilapia triggered by the divergent metabolic responses in the two species to the commonly severe cold.

### The same cooling scheme induces differential apoptosis progression in the gills of zebrafish and tilapia

**Figure 5:**
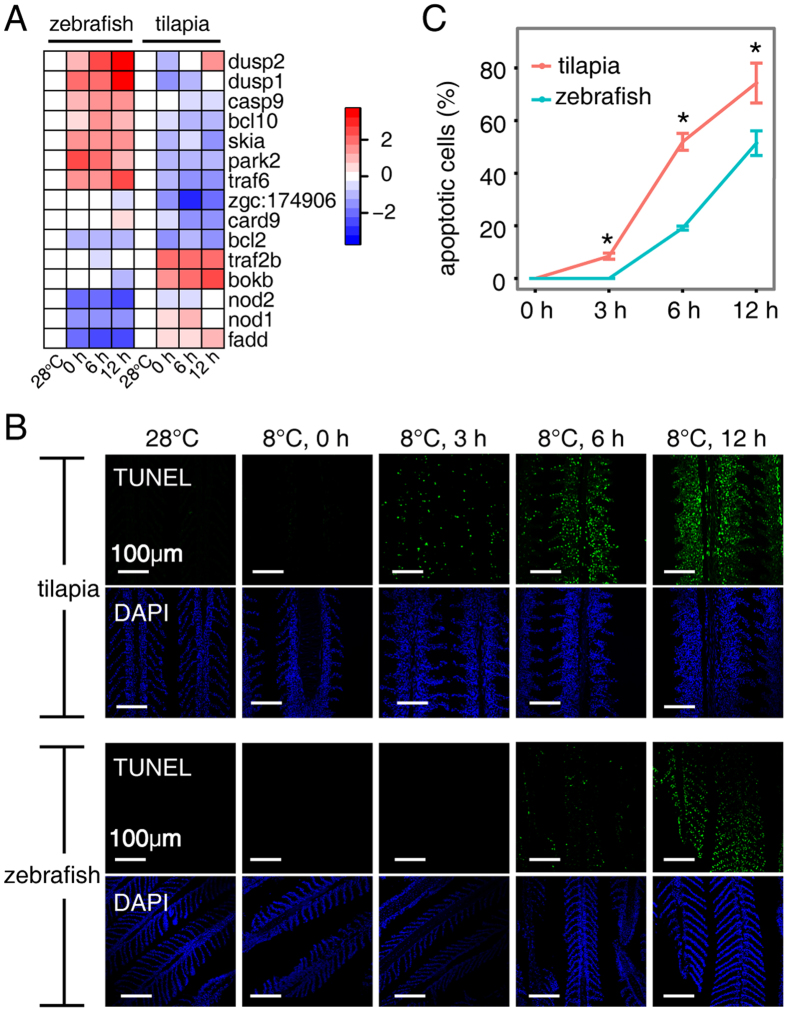
Inter-specific divergence in apoptosis. (**A**) Hierarchical clustering of zebrafish/tilapia divergently expressed genes contained in the GO term “regulation of apoptotic process”. Log_2_ transformation of gene fold change is indicated by the color-coded scale. (**B**) TUNEL assays in the gills of two fishes, indicating an earlier onset and more aggravated apoptosis in tilapia than in zebrafish at the same cold temperature. The nucleus was counterstained with DAPI. Scale bar is 100 μm. (**C**) Increased apoptotic cell populations in the gills of tilapia and zebrafish over time at 8 °C. Significant differences between the two fishes are indicated by an asterisk (Student’s t test, *P* < 0.05) and are based on at least three biological replicates, with each replicate having at least 3 individuals at each time point.

We next assessed the functional consequences of expression divergence between zebrafish and tilapia. Apoptosis were significantly over-represented in the zebrafish/tilapia divergently expressed genes at the later time points (Fig. 3E), and many enriched GO terms are associated with apoptosis, including “regulation of apoptotic process”, “cellular response to DNA damage stimulus” and “DNA repair”. Figure 5A shows a heat map for 15 zebrafish/tilapia divergently expressed genes within the GO category “regulation of apoptotic process”, including two dual specificity phosphatases (*Dusp1* and *Dusp2*), two TNF receptor associated factors (*Traf6* and *Traf2b*), two nucleotide-binding oligomerization domain containing genes (*Nod1* and *Nod2*), and 9 other genes (Fig. 5A). We confirmed the expression changes of *Dusp1* by quantitative PCR (qPCR) (Fig. S2). The prevalence of gene expression divergence in apoptosis suggests that cold may differentially affect apoptosis progression in the gill cells of the two fishes.

To verify this hypothesis, we examined the signals of apoptosis in the gills of tilapia and zebrafish exposed to 8 °C at 3 h intervals by terminal deoxynucleotidyl transferase dUTP nick end labeling (TUNEL) staining. As predicted, we observed a difference in the timing of apoptosis onset in the gills of the two fishes. Apoptotic cells were initially detectable at the 3 h time point in tilapia gill but were absent in zebrafish gill (Fig. 5B). With continued cold stress from prolonged 8 °C exposure, the proportion of apoptotic cells in the tilapia gill was always greater than that of the zebrafish gill at any time point (Fig. 5C). When death ensued in the tilapia (at 12 h at 8 °C), approximately 74% of gill cells had undergone apoptosis, compared with 51% in zebrafish gill (Fig. 5C). At this time, the zebrafish began to lose body equilibrium. To evaluate whether cold-induced damage also happened to other tissue types, we examined apoptotic signals in brain, liver, kidney, spleen and muscle at 12 h exposure to 8 °C. Apoptotic cells were detected only in kidneys of the two fishes with significantly less intensity (Fig. S4). Inter-species differences in apoptosis are likely the outcomes of the different LTLs between species, as excessive apoptosis is a sign of functional impairment leading to physiological disturbances. Taken together, the results suggest that the extent of apoptosis in gill correlates with the LTL of the fish.

### Different in *cis*- and *trans*- environment contributed to divergently apoptotic regulation between zebrafish and tilapia

To reveal the potential transcriptional regulation underlying the differential apoptosis in the gills of the two fishes, we surveyed transcription factor binding sites (TFBSs) within the proximal promoters of the zebrafish/tilapia divergently expressed genes that were associated with apoptosis. Two hundred and five TFBSs in the JASPAR database were used for scanning the upstream 1 kb from the transcription start sites of 65 genes within the GO term “regulation of apoptotic process”. We identified 10 transcription factors, including 2 signal transducer and activator of transcription (STAT) proteins, NF kappa B1 (Nfkb1), B-cell lymphoma 6 (Bcl6) and 6 other transcription factors, whose binding sites were enriched in the promoters of the zebrafish/tilapia divergently expressed genes (Table 1). This finding suggests a potential contribution of the TFBSs to differential apoptosis regulation.

**Figure 6:**
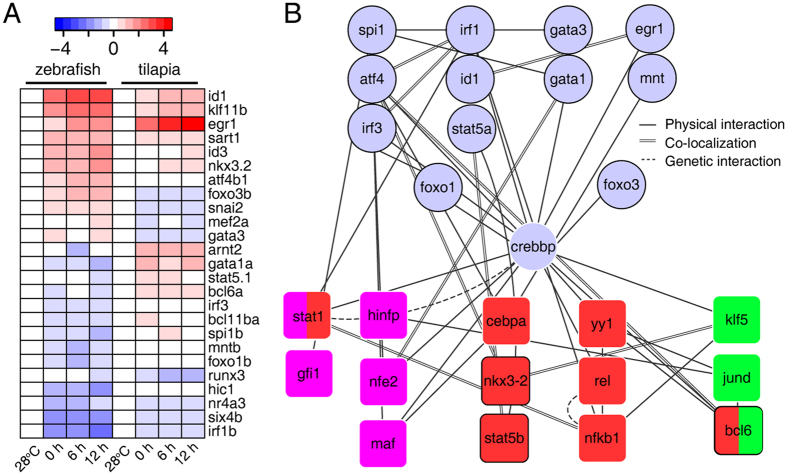
Regulatory networks showing the inter-specific divergence in *cis*- and *trans*- regulation of apoptosis. (**A**) Hierarchical clustering of 25 zebrafish/tilapia divergently expressed transcription factors that were apoptosis-related. (**B**) Protein-protein interaction network for zebrafish/tilapia differently expressed and TFBS-enriched transcription factors in the metabolism, FoxO and apoptosis pathways. Quadrate nodes denote the transcription factors whose binding sites were enriched in the promoter of zebrafish/tilapia divergently expressed genes. The pathways to which the enriched transcription factors belong were indicated by different colors, with pink for metabolic pathways, red for apoptosis, and green for FoxO signaling pathway. The circles with black border represent the zebrafish/tilapia divergently expressed apoptosis-related genes, while circle without the black border indicates an intermediate protein of the network, in which significantly expressional divergence between zebrafish and tilapia was not detected in this study. Protein-protein interaction information was retrieved from the literature using GeneMANIA. The types of protein-protein interactions are represented by different types of lines, with the solid lines representing the physical interactions, parallel lines representing co-localization and dots representing genetic interactions. According to definitions in GeneMANIA, “Physical interaction” refers to an experimentally validated protein-protein interaction, and “Co-localization” refers to two proteins that co-localize in the cell. “Genetic interaction” indicates that the effects of one gene are modified by another gene identified through mutation analysis.

To further identify the potential trans-factors involved in the differential apoptosis regulation, we screened for transcription factors that are apoptosis-related (defined by the description of GO term, see Methods). We defined 83 apoptosis-related transcription factors, of which, a total of 25 were divergently expressed between zebrafish and tilapia (Fig. 6A). Interestingly, *Bcl6, Stat5a, Stat5b,* and *Nkx3.2* are zebrafish/tilapia divergently expressed gene (Fig. 6A and Table S3) whose binding sites are enriched in the promoters of zebrafish/tilapia divergently expressed genes within the GO term “regulation of apoptotic process”, suggesting the involvement of these transcription factors in the divergent regulation of apoptosis between zebrafish and tilapia. We used the same methodology to identify the transcription factors in the metabolism pathways, insulin signaling pathway and FoxO signaling pathway, whose binding sites were enriched in promoters of the zebrafish/tilapia divergently expressed genes. The effort resulted in 6, 5 and 3 transcription factors in the three pathways respectively (Table S4). No enriched transcription factor binding sites were detected in the genes of the “Pyrimidine metabolism” pathway.

To further investigate the interaction patterns of the apoptosis-related TFs with transcription factors from other KEGG pathways whose binding sites were enriched in the divergently expressed genes, we obtained evidence of protein interactions (genetic interactions, physical interactions or co-localization) between the transcription factors. Based on the records in GeneMANIA database, we constructed a regulatory network and found that 52% (14/25) of apoptosis-related transcription factors and 18 TFBS-enriched transcription factors were interconnected (Fig. 6B). In addition to the direct interactions, some of the transcription factors interact through common partners, for example, FoxO3 could interact with Bcl6 and other factors through Crebbp.

## Discussion

Several studies on the transcriptional responses under cold stress in fish gill have been documented^18^,^19^. The transcription factors regulating cold responses and the molecular mechanisms leading to the varied LTLs among various fish species are still elusive. In this study, we compared the transcriptional responses in the gills of zebrafish and tilapia when exposed to a lethally cold temperature. We identified the gene expression divergence between the two fish species and found that 5 pathways, including metabolic pathways, insulin signaling pathways, pyrimidine metabolism, apoptosis and FoxO signaling pathway, were differentially regulated in sequence in the duration of lethally cold-exposure. We verified the cellular consequence of the apoptosis-biased transcription divergence by examining the presence of apoptotic cells in the gills of the two fishes and found a clear disparity in the onset and progression of apoptosis in this tissue that directly contacts the environment and is critical in stress responses (Fig. 4). Further close examination of zebrafish/tilapia divergently expressed genes within the 5 KEGG pathways revealed enriched binding sites for 22 transcription factors in their proximal upstream regions. In addition, more than 86% (19/22) transcription factors could potentially interact with each other, suggesting that multiple transcription factors may contribute to the divergent regulation in the two fish species which contribute to the differential apoptosis in the two fishes.

It is also interesting to note that no other tissue except that of the kidney had apoptosis signals that could be detected in either fish. Because apoptosis can be used as an indicator of cellular damage^35^,^36^, it is plausible to hypothesize that the gill tissue in a fish is the most cold-sensitive tissue. Although more solid experimental evidence is needed, the current study suggests that the degree of functional impairment in the gill may set the threshold for unrecoverable physiological failure leading to mortality. This hypothesis is supported by the correlation between the severity of physiological disturbances and the proportion of apoptotic cells in the gill at the lethally cold temperature. In tilapia, the fish fell into coma when the signals of apoptosis began to appear and died when ~75% of the gill cells underwent apoptosis (Fig. 4C). A similar correlation was also detected in zebrafish, but delayed, presumably because its LTL is lower than tilapia’s. To further support this link, we exposed the grass carp *Ctenopharyngodon idella*, a species tolerant to a wide range of temperature and that actively swims at 8 °C and examined signals of apoptosis in the gill. As predicted, apoptotic cells were only sporadic (<1% of total cells) in the gill after 12 h of 8 °C exposure (Fig. S5). Thus, the intensity of cold-induced apoptosis in gill is closely correlated with the differential LTL in these three fishes that are evolutionary distant. The genetic factors and the interacting networks contributing to the differential apoptosis regulation among the fishes may play roles in setting the lower boundary of cold tolerance, which can be further validated in the future.

The time course in which the species-divergent KEGG pathways appeared provided us important clues to the possible trigger and the signal pathways lead to irreversible tissue damage in the fish exposed to LTL. In this study, we clearly revealed that several metabolic pathways including the insulin signaling pathway are the early diverged pathways, followed with pyrimidine metabolism and apoptosis, and at last, apoptosis and FoxO signaling pathways. The detected divergence in apoptosis regulation at 6 h is nicely correlated with the largest difference in the percentage of apoptotic cells in the gills of the two species at this time point (Fig. 5C), suggesting that our approach is sensitive in uncovering the diverged phenotypic differences in the two species. Based on the time course of divergent KEGG pathways and their interrelationship (Fig. 4), it is reasonable to propose that dysregulated metabolic pathways in the lethally cold temperature is likely the trigger of the downstream pathways such as apoptosis and FoxO signaling pathways which lead to the differential outcome in apoptosis, and therefore contributed to differential LTLs in the fishes (Fig. 4).

An intriguing finding in this study is that genes involved in repair and regeneration signaling are divergently expressed at 12 h, suggesting an attempt for tissue repair even when a greater portion of the cells in the tilapia gill had undergone apoptosis. This situation may occur because cells in the gill might be heterogeneous in terms of their capacity in cold tolerance, and the fate of the cells facing the severe cold is determined by the dynamics of the factors regulating cell repair and cell apoptosis. At 12 h, we found that the FoxO signaling pathway was differentially regulated. FoxO signaling regulates cell survival, apoptosis, and DNA repair^37^, thus play important roles in tissue homeostasis regulation in stress conditions^33^. Under lethally cold temperature, DNA repair and apoptosis may both be induced, but apoptosis overwhelmed in tilapia, such that the majority of the cells underwent apoptosis at 12 h in this fish. The final outcome is determined by the percentage of the cells undergo irreversible damage (apoptosis). Taken together, we propose that the divergently expressed genes in metabolism pathways, apoptosis and FoxO signaling are collectively important in determining LTL of fish. In addition to the genetic factors, acclimation (the history of thermal experience of a fish) may also influence cold tolerance^38^,^39^. For example, the limit of low temperature tolerance can be extended for zebrafish after acclimation at 20 °C^40^.

The expression pattern of several genes in our constructed network (Fig. 6B) has been documented in previous studies to be induced by cold stress. For example, the expression of inhibitor of DNA binding 1 (*Id1*) is markedly increased in cold-acclimated zebrafish^19^. The activation of activating transcription factor 4 (Atf4) is important for cold adaptation in ground squirrels^41^ and mice^42^, and its expression is increased at cold temperatures in zebrafish^19^. The pervasive induction of key transcription factors, such as Id1 and Atf4, could be regarded as markers of cold response and adaptation. Notably, the expression of *Atf4* and *Id1* was divergently expressed between the zebrafish and tilapia. We observed strong induction of *Atf4* and *Id1* in zebrafish but not in tilapia (Fig. 6A). In a previous work using zebrafish as a model^19^, we found that at normal temperature, Bcl6, a known transcriptional repressor^43^ physically interacts with Jun, a factor critical in stress responses^44^. Interestingly, the Bcl6-Jun interaction is temperature-dependent, as the amount of the Bcl6/Jun complex decreases with temperature reduction^19^, suggesting the involvement of Bcl6 in cold responses in zebrafish. Bcl6 also directly or indirectly interacts with other divergently expressed TFs such as Atf4, Spi1, Arntt2, FoxO3 and others (Fig. 6B). The identification of these factors and their relationship facilitates our future investigations of molecular mechanisms of cold tolerance in fishes.

Regulatory elements in promoters play a major role in the genesis of new phenotypes^45^,^46^. Transcriptome divergence among species is attributable to different distribution of TATA box^25^, CpG island^47^ and transcription factor binding sites^24^ in the promoter regions of genes. We found that genes involved in regulation of apoptosis were significantly enriched with binding sites for Stats, Bcl6, Nfkb1, Nkx3-2, Cebppa and others, and the divergently expressed genes in the metabolic pathways are enriched with binding sites for Nfe2, Maf, Stats, Hinfp and Gfi1. Binding sites for rora, hnf4g and klf5, jund, bcl6 are enriched in the divergently expressed genes in the insulin and FoxO signaling pathways, respectively. The protein interaction network (Fig. 6B) indicates that transcription factors from these pathways are interconnected. Hinfp of the metabolic pathway physically interacts with Jund of the FoxO pathway. More importantly, multiple transcription factors in these two pathways commonly interact with crebbp which interact with FoxO1, FoxO3 and transcription factors of the apoptosis pathways. Some transcription factors are known to involve in multiple signaling pathways, for example, STAT1 involves in metabolism and apoptosis, while bcl6 in both FoxO and apoptosis pathways and these factors themselves are divergently expressed between zebrafish and tilapia in cold stress, and thus are likely play important roles in regulating the cold-induced apoptosis. It is yet to known which transcription factor(s) in the interaction network play(s) significant roles in producing the differential apoptotic intensities in the gills of the two fishes and therefore contribute substantially to LTL determination in fish.

In summary, we performed a transcriptome comparison between zebrafish and tilapia gills under cold stress. We investigated the expression divergence of orthologous genes between these species. We identified multiple regulatory pathways which are divergently expressed between the two species in the course of cold exposure. Moreover, we validated there considerable divergence in the onset and progression of apoptosis in the gills of the two fishes. We suggest that this regulatory divergence is likely to be one contributing factor to the different LTLs of the two species under cold stress. The capacity for differential regulation of apoptosis is enriched with the biased distribution of the binding sites for certain transcription regulators (e.g., Stats, Bcl6 and Nfkb) in the gene promoters. Due to the limited sample size and time points we have surveyed, the current list of divergently expressed genes and the genetic network is probably not complete. Nonetheless, the current work provides insights into the genetic basis for the different LTLs in various fishes.

## Methods

### Fish and cold exposure

Tilapia (*O. niloticus*) were reared at 28 °C for at least 5 generations under laboratory conditions. Zebrafish (*D. rerio*) were maintained at 28 °C in the laboratory for at least 10 generations. Both fish species were raised in fresh water and fed the same commercial diet at 4% of their body weight daily. For cooling, six-month-old tilapia and zebrafish were subjected to cold in a temperature-adjustable cultivation chamber (Ningbo Jiangnan Instrument Factory, Ningbo, China). The cooling scheme was similar to that in a previous study^19^. The temperature graded declines from 28 °C to 18 °C in 12 h at a rate of ~0.85 °C/h. After 12 h acclimation at 18 °C, the temperature continuously dropped to 8 °C in 12 h and was maintained at 8 °C. Tilapia experienced a loss of equilibrium and entered a coma within half an hour after the temperature decreased to 8 °C. At four temperature/time points (28 °C, 8 °C/0 h, 8 °C/6 h and 8 °C/12 h), tilapia and zebrafish were anaesthetized and killed immediately by decapitation to collect gill tissues. Branchial filament was carefully excised under the microscope. To ensure the consistency, individuals of tilapia and zebrafish were sampled in parallel at the determined time points. At each time point, the timings of sampling were identical between the two species to minimize circadian fluctuation. Each tilapia sample comprised a single fish, whereas 10 zebrafish were pooled in equal proportions to generate RNA amounts comparable to those obtained from one tilapia, due to the small size of the zebrafish gill. At each temperature/time point, three biological replicates were collected in both fish species. For RNA-Seq, twenty-four samples (2 species × 3 time points × 3 biological replicates and three replicates for control) were sequenced. The experiments were conducted according to the principles expressed in the “Guide for the Care and Use of Laboratory Animals” published by the National Research Council of the National Academies. The protocols used were approved by the Ethics Committee for the Use of Animal Subjects of Shanghai Ocean University.

### RNA preparation

Total RNA was extracted using TRIzol Reagent according to the manufacturer’s protocol (Invitrogen, Carlsbad, CA). RNA degradation and contamination were monitored on 1% agarose gels. The quality of RNA was assessed by measuring the RNA Integrity Number (RIN) using a Bioanalyzer Chip RNA 7500 series II (Agilent). Samples with RIN greater than 8.0 were used for sequencing. The concentration of total RNA was determined with a Qubit fluorometer (Life Technologies).

### RNA sequencing

Three micrograms of RNA from each sample was used to prepare the mRNA-Seq library with the TruSeq RNA Sample Prep Kit (Illumina) following the manufacturer’s instructions. Proper index codes were used to attribute sequences to individual samples. Briefly, poly(A)+ RNA was purified and fragmented using divalent cations at elevated temperature. RNA fragments were converted to cDNA using random primers, followed by second-strand cDNA synthesis and end repair. Illumina PE adaptors were attached to the cDNA ends. Fragments that were approximately 300 bp in length was extracted from a 2% low-range ultra-agarose sizing gel. Adaptor-tagged cDNA fragments were enriched using the manufacturer’s cocktail and 10-cycle PCR. The library quality and insert length were checked using the DNA High Sensitivity DNA Kit (Bioanalyzer 2100, Agilent) to ensure the proper insert size of 300–500 bp. The libraries were diluted to 10 pM, and equal amounts of 8 distinctively indexed libraries were mixed and subjected to 109 cycles of paired-end (2 × 109 bp) sequencing on one lane of an Illumina HiSeq 2000 system. A total of three lanes were used to sequence the entire sample set.

### The annotation databases

The reference genome sequences and gtf files were downloaded from Ensembl release 78 (http://www.ensembl.org/info/data/ftp) for both fishes. The “one-to-one” orthologous gene pairs between zebrafish and tilapia and GO annotations associated with Ensembl gene id numbers were obtained from Biomart in Ensembl^48^.

### Pre-processing and mapping of RNA-Seq reads

Trimmomatic 0.32 49 was used to remove the adaptor sequence and low-quality bases from the raw reads. First, the parameter in ILLUMINACLIP was set to 2:30:10 to remove the adaptor sequences from the raw reads. The 2 bases from the start and the end of the read were then removed. The reads were trimmed via sliding window trimming when the average quality within 4 bases fell below 20, and a post-trimming length of 50 bases or longer was enforced. Finally, the trimmed reads that were paired were retained for mapping. TopHat 2.0.13 50 was used to map the reads to the reference genomes. Default values were set for the parameters of TopHat read mapping. The reads from each sample were mapped separately.

### Expression quantification, differentially expressed genes and zebrafish/tilapia divergently expressed genes

SAMTools^51^ was used to first sort the bam files of the aligned reads by read name. HTSeq-count^52^ was then applied to count the number of reads that were mapped to the genes. The read counts of each sample were imported into the R package edgeR^53^ for a differential expression analysis. We compared the gene expression level at low temperature/time points (8 °C/0 h and 8 °C/12 h) with that at control temperature (28 °C). Genes with a fold change greater than 2 and an FDR less than 0.05 were considered differentially expressed. A GLM approach was used to identify zebrafish/tilapia divergently expressed genes for the selected time points at 8 °C. In this approach, two factors, species (zebrafish and tilapia) and temperature/time point (28 °C, 8 °C/0 h and 8 °C/12 h), were combined into one factor that consisted of 6 levels (tilapia: 28 °C, tilapia: 8 °C/0 h, tilapia: 8 °C/12 h, zebrafish: 28 °C, zebrafish: 8 °C/0 h, and zebrafish: 8 °C/12 h). We eliminated the effect of the initial expression patterns by normalizing the gene expression level at 8 °C to that at 28 °C. We then identified orthologous genes that were divergently expressed between the two fishes at 0 or 12 h. For example, we could identify zebrafish/tilapia divergently expressed genes at 8 °C/0 h using the following contrast in R script: “contrast = (tilapia: 8 °C/0 h − tilapia: 28 °C) − (zebrafish: 8 °C/0 h − zebrafish: 28 °C)”. At 0 or 12 h at 8 °C, genes with a fold change greater than 2 and an FDR less than 0.05 were considered zebrafish/tilapia divergently expressed genes.

### GO enrichment analysis

To identify functional categories associated with cold stress, the GO annotations of tilapia and zebrafish were downloaded from the Ensembl database. An enrichment analysis was performed via a hypergeometric test. The P value was calculated using the following formula:


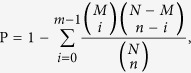


where N is the total number of genes, n is the total number of zebrafish/tilapia divergently expressed genes, M is the number of genes annotated to a certain GO term, and i is the number of zebrafish/tilapia divergently expressed genes annotated to a certain GO term. GO terms with P value below 0.05 were considered enriched. All statistical calculations were performed in R.

### Validation of RNA-Seq results by reverse transcription qPCR

Two micrograms of RNA from each sample was reverse-transcribed to cDNA using an RT-PCR kit (TaKaRa). qPCR was performed using SYBR Green Master Mix following the manufacturer’s protocol (Roche). *β-actin* was used as the internal control because the RNA-Seq data indicated that its expression remained largely constant during temperature treatment. The primers are listed in Table S6.

### Immunofluorescent staining

Paraffin sections were used for immunofluorescence staining. The tissue sections were deparaffinized in xylene and subsequently treated with sodium citrate buffer (pH 6.5) at 95 °C for 5 minutes. TUNEL staining was performed using a commercially available kit (TUNEL FITC Apoptosis Detection kit, Vazyme, A11103). The tissue sections were then counterstained with DAPI (500 ng/ml) for 5 minutes and analyzed under a laser confocal microscope (LSM 710; Carl Zeiss, Jena, Germany).

### Enrichment analysis of TFBSs in the proximal promoters of divergently expressed genes

We identified zebrafish and tilapia orthologous genes using the Ensembl database, and canonical Ensembl transcripts linked to each orthologous genes were obtained. We retrieved the information of transcription start sites (TSSs) at the first base of the 5′ UTR of each canonical transcript in Ensembl. By doing this, 14,475 pairs of TSS features were defined. The weight matrixes of TFBSs were obtained from the JASPAR^54^ database. The presence of a TFBS was determined by the motif-finding software FIMO^55^ with the default threshold (P < 0.0001). The occurrence of TFBSs was assessed on both strands of the 1 kb upstream of TSS in each gene. The motif-containing orthologous genes were defined as those whose promoters contain the motif in any one of zebrafish and tilapia. Fisher’s exact test was used to test against the null hypothesis.

### Apoptosis-related transcription factor definition

We first identified the transcription factors based on the records on the DBD database^56^. We defined 551 transcription factors in our study. Currently, there is little information associated with apoptosis in the GO annotation of zebrafish, which prompted us to use the GO annotation information for human records. “Apoptosis-related transcription factors” were defined as those whose GO annotation was matched to “apoptosis” or “apoptotic”. Based on the GO annotation in human, we defined 83 apoptosis-related transcription factors in our analysis.

## Additional Information

**Accession codes:** Sequence data have been deposited at the GEO of NCBI, under accession number GSE69965.

**How to cite this article**: Hu, P. *et al*. Transcriptome comparison reveals a genetic network regulating the lower temperature limit in fish. *Sci. Rep.* **6**, 28952; doi: 10.1038/srep28952 (2016).

## Supplementary Material

Supplementary Information

## Figures and Tables

**Table 1:** Enrichment analysis of TFBSs in promoters of zebrafish/tilapia divergently expressed genes within the GO term “regulation of apoptotic process.”

TF	Zebrafish/tilapia divergently expressed orthologues		Zebrafish/tilapia non divergently expressed orthologues		Odds ratio	P value
	Present	Absent	Present	Absent		
STAT1	15	9	8	33	6.63	9.84E-04
NFKB1	13	11	8	33	4.74	5.98E-03
Bcl6	14	10	10	31	4.23	8.44E-03
Esrrb	10	14	6	35	4.06	1.95E-02
CEBPA	16	8	15	26	3.40	2.31E-02
REL	9	15	5	36	4.21	2.73E-02
YY1	12	12	9	32	3.48	2.83E-02
Stat5a::Stat5b	12	12	9	32	3.48	2.83E-02
Nkx3-2	10	14	7	34	3.40	4.16E-02
ESRRA	11	13	8	33	3.42	4.61E-02

The present or absence of a TFBS motif was identified by FIMO. Odds ratio quantifies the effect of the presence of a motif from this class on the odds that it would be divergently expressed. P values were calculated using Fisher’s exact test against the null hypothesis (a no-biased distribution of TFBS in the promoters of the genes of interest).
