# Supplementary material for: Transcriptome comparison reveals a genetic network regulating the lower temperature limit in fish
Source: Sci Rep. 2016 Jun 30;6:28952. doi: 10.1038/srep28952 (PMC4928090; doi:10.1038/srep28952)
Supplement: Supplementary Information [file srep28952-s1.pdf]

Transcriptome comparison reveals a genetic network regulating the lower temperature limit in fish

Peng Hu[1], Mingli Liu[1], Yimeng Liu[1], Jinfeng Wang[1], Dong Zhang[1], Hongbo Niu[1], Shouwen Jiang[1], Jian Wang[1], Dongsheng Zhang[1], Bingshe Han[1], Qianghua Xu[2], and Liangbiao Chen[1,*]

[1]Key Laboratory of Aquacultural Resources and Utilization, Ministry of Education, College of Fisheries and Life Sciences, Shanghai Ocean University, Shanghai, 201306, China
[2]Key Laboratory of Sustainable Exploitation of Oceanic Fisheries Resources, Ministry of Education, College of Marine Sciences, Shanghai Ocean University, Shanghai, 201306, China
[*] Correspondence: Liangbiao Chen, Shanghai Ocean University, 999 Huchenghuan Road, Lingang New City, Shanghai 201306, P.R. China. E-mail: lbchen@shou.edu.cn

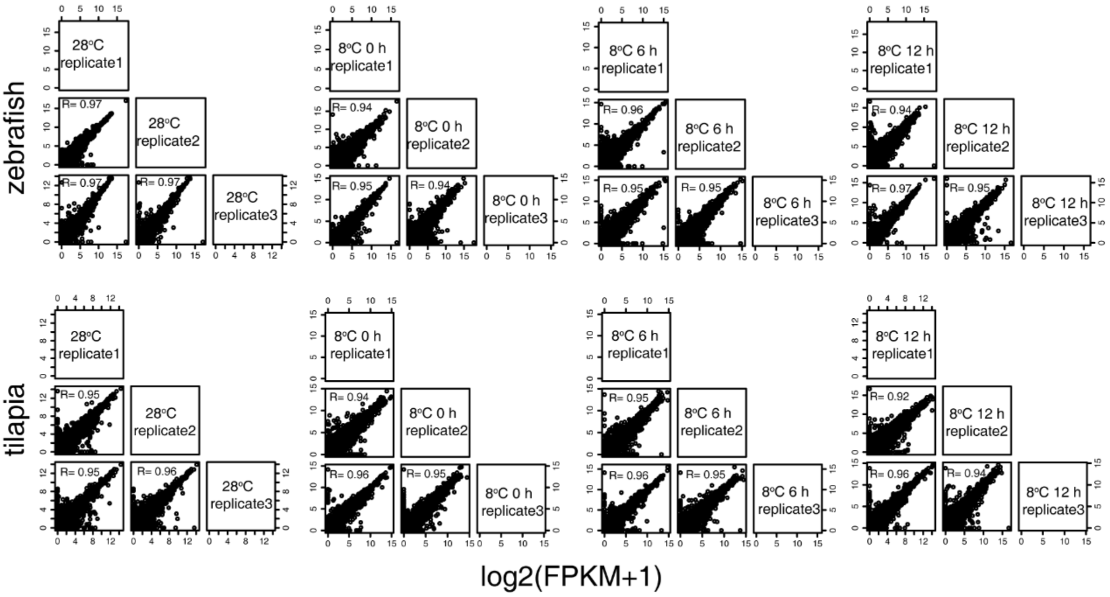

Figure S1. Scatter plot showing gene expression value (FPKM) between biological replicates. Pearson correlation coefficient (*R*) is indicated.

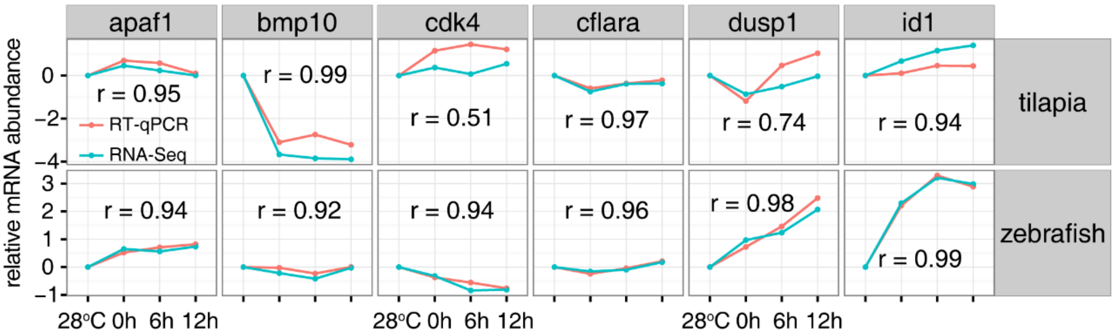

Figure S2. The expression patterns of 6 genes under cold treatments measured by RT-qPCR in comparison with those deduced from the RNA-seq results. The Pearson correlation coefficient (r) between RT-qPCR and RNA-seq was indicated.

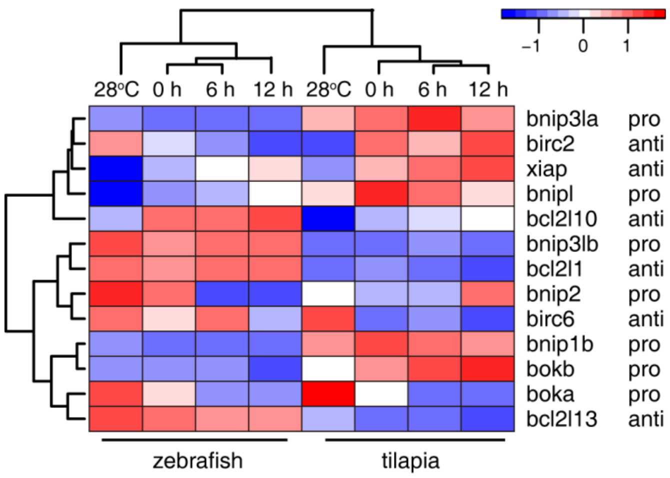

Fig. S3 Clustering analysis of changes in gene expression for apoptosis-related genes in the zebrafish and tilapia transcriptome. Genes that were assigned to anti-apoptosis and pro-apoptosis, which was indicated in the right. The colors represents relative expression changes. The dendrograms represent the differences of gene expression at each time point.

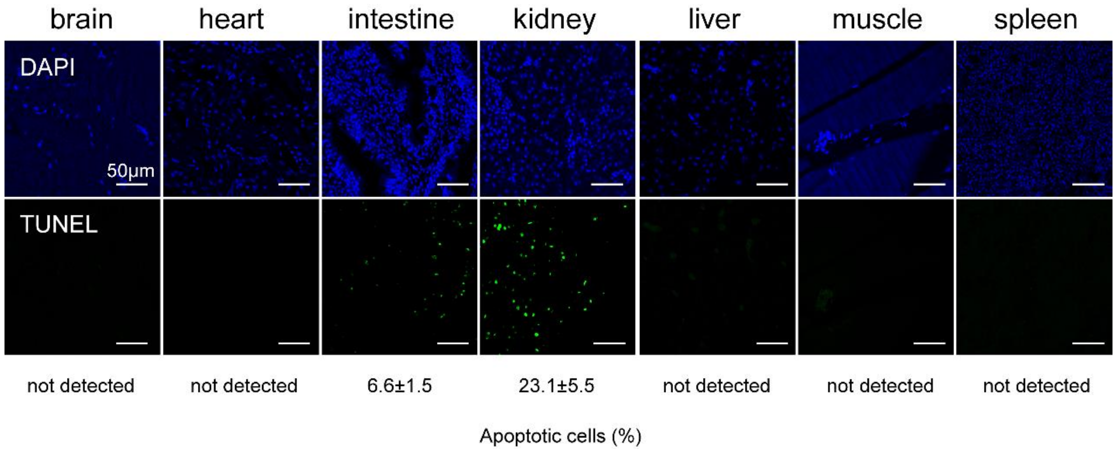

Figure S4. Apoptosis analysis of other 7 tissues at 8°C/12 h by TUNEL. The nucleus was counterstained with DAPI. Scale bar is 50 μm. The proportion apoptotic cells (Mean ± SD) is indicated in the below and are based on at least three biological replicates, with each replicate having at least 3 individuals.

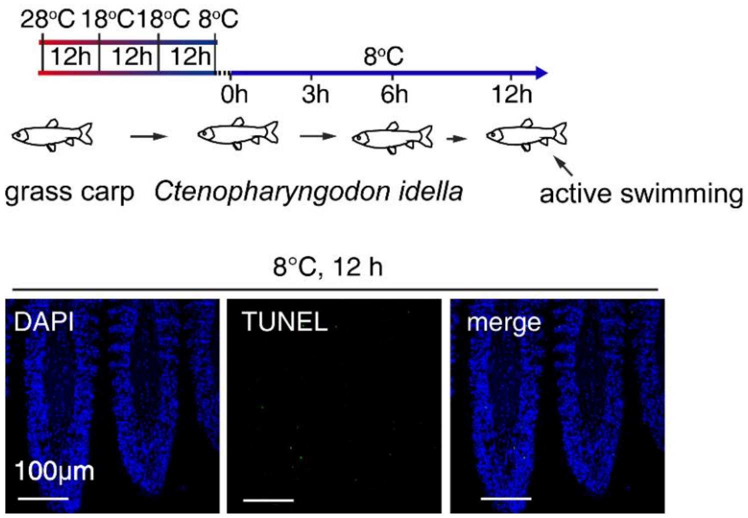

Figure S5. Apoptosis analysis of grass carp *Ctenopharyngodon idella* gill at 8°C/12 h by TUNEL. The nucleus was counterstained with DAPI. Scale bar is 100 μm.

Table S1. The numbers of raw, clean and mapped RNA-seq reads from each sample.

Group	replicate	species	condition	raw reads	trimmed reads	mapped reads
1	1	tilapia	28°C	11,941,194	10,530,042	8,044,543
1	2	tilapia	28°C	14,131,725	12,499,448	9,409,733
1	3	tilapia	28°C	14,295,169	12,576,255	9,660,826
2	1	tilapia	8°C/0 h	15,675,972	13,806,819	10,653,212
2	2	tilapia	8°C/0 h	13,427,850	11,703,436	9,119,121
2	3	tilapia	8°C/0 h	9,028,886	8,222,948	6,337,739
3	1	tilapia	8°C/6 h	17,026,820	15,058,697	11,876,697
3	2	tilapia	8°C/6 h	14,856,128	12,806,119	9,777,828
3	3	tilapia	8°C/6 h	11,062,991	9,796,104	7,873,444
4	1	tilapia	8°C/12 h	15,215,259	13,141,915	10,381,896
4	2	tilapia	8°C/12 h	12,426,254	10,826,850	8,602,261
4	3	tilapia	8°C/12 h	13,247,555	12,048,912	9,243,886
5	1	zebrafish	28°C	13,155,052	11,152,742	8,819,536
5	2	zebrafish	28°C	12,023,477	10,697,001	8,384,085
5	3	zebrafish	28°C	14,927,030	13,190,698	10,327,914
6	1	zebrafish	8°C/0 h	15,437,052	13,625,528	10,843,720
6	2	zebrafish	8°C/0 h	15,202,780	13,494,226	10,765,579
6	3	zebrafish	8°C/0 h	12,192,505	11,178,979	8,979,117
7	1	zebrafish	8°C/6 h	14,713,329	13,017,569	10,639,129
7	2	zebrafish	8°C/6 h	14,692,433	12,781,203	10,378,855
7	3	zebrafish	8°C/6 h	15,122,071	13,106,798	10,984,988
8	1	zebrafish	8°C/12 h	12,886,557	11,377,284	9,021,792
8	2	zebrafish	8°C/12 h	15,751,804	13,459,976	10,488,727
8	3	zebrafish	8°C/12 h	16,793,025	14,594,684	12,146,405
Total	24 samples			335,232,918	294,694,233	232,761,033

Table S2. List of cold-responsive genes (see Dataset 1)

Table S3. List of zebrafish/tilapia divergently expressed genes (see Dataset 2)

Table S4. Enrichment analysis of TFBS in promoters of zebrafish/tilapia divergently expressed genes within the metabolic pathways, insulin signaling pathway, and foxo signaling pathway.

TF	divergently expressed orthologues		non divergently expressed orthologues		Odds Ratio	Pvalue	Pathway
	present	absent	present	absent			
Spz1	11	14	12	62	3.99	1.17E-02	Insulin signaling pathway
NKX3-1	12	13	17	57	3.06	2.32E-02	Insulin signaling pathway
HNF4G	15	10	25	49	2.91	3.28E-02	Insulin signaling pathway
Nobox	7	18	7	67	3.66	4.10E-02	Insulin signaling pathway
RORA_1	12	13	19	55	2.64	4.76E-02	Insulin signaling pathway
Bcl6	16	14	18	52	3.26	1.11E-02	FoxO signaling pathway
KLF5	21	9	30	40	3.08	1.65E-02	FoxO signaling pathway
JUND	17	13	22	48	2.82	2.51E-02	FoxO signaling pathway
Gfi1	102	78	226	296	1.71	2.38E-03	Metabolic pathways
NFE2::MAF	84	96	178	344	1.69	3.16E-03	Metabolic pathways
Stat4	84	96	180	342	1.66	4.27E-03	Metabolic pathways
E2F6	62	118	132	390	1.55	2.04E-02	Metabolic pathways
STAT1	70	110	155	367	1.51	2.62E-02	Metabolic pathways
HINFP	27	153	47	475	1.78	3.38E-02	Metabolic pathways

Table S5. List of the primers used in this study

Gene		zebrafish	tilapia
bmp10	Forward	GAGTTAGCATCTCGACAGGTTTAT	GACAACCCTAGAGGGCATAAAT
	Reverse	CGTGGTCCCATAGTCTGATTTC	GCAGGATGGAGTATGTCAAGAA
dusp1	Forward	GCTGGGTTTGTTTCGTTCATC	CTCAGAGTAGATGTGCCAGAAAG
	Reverse	CTCCAACATATCCCGAAGTGAG	GGCTTCATGATGTCTCGTAAGG
apaf1	Forward	CATTTGTGGCTCTGGGATCT	CGACAATATCGGCATCCTCTAC
	Reverse	GCAGTGGTGAACAGTCTTAGT	GGCGTCTGTTTCTGTCATCT
cdk4	Forward	AGGACGGATCAGGAGACTAAAG	TTCACTCTAACCGCGTGATG
	Reverse	GCTGGAACTTTCTCCAGGTATG	AGATCCTGGCAAGTCCAAAG
cflara	Forward	CTCTCTTGGGTCTGAAGGAAAC	GATGTCCACCAGTATCTGAGTTC
	Reverse	TGATGAGGCAGCAGACAAAG	GATGCAGCACACAAAGCTATC
id1	Forward	CTCGCTTCAGCTATTCCTCTTT	TGACAGGATCATGTGTCGTTAAG
	Reverse	CAACATCCTCTCCTCCAACTTT	CTTGATGCGGTTCGAGAAGT
actb	Forward	GATCTGGCATCACACCTTCTAC	GATCTGGCATCACACCTTCTAC
	Reverse	TCTTCTCCCTGTTGGCTTTG	TCTTCTCTCTGTTGGCTTTGG
